# Supplementary material for: Vascular cell-adhesion molecule 1 (VCAM-1) regulates JunB-mediated IL-8/CXCL1 expression and pathological neovascularization
Source: Commun Biol. 2023 May 13;6:516. doi: 10.1038/s42003-023-04905-z (PMC10183029; doi:10.1038/s42003-023-04905-z)

Supplementary Figure 1: Scans of immunoblots presented in Figure 1b, 1c, 1e & 1f.

Figure 1b

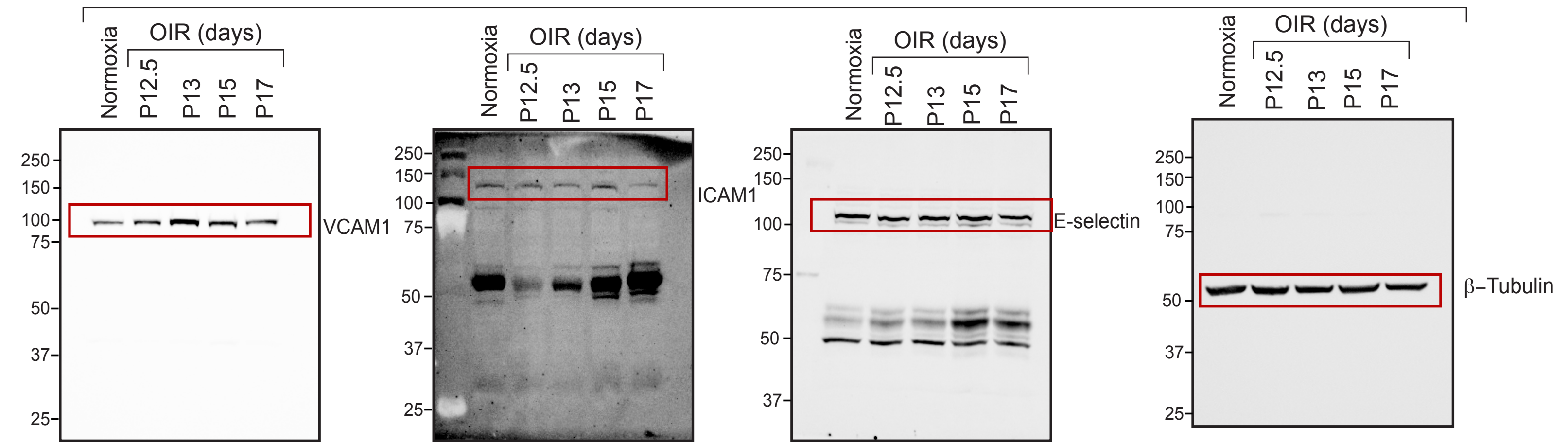

Figure 1c

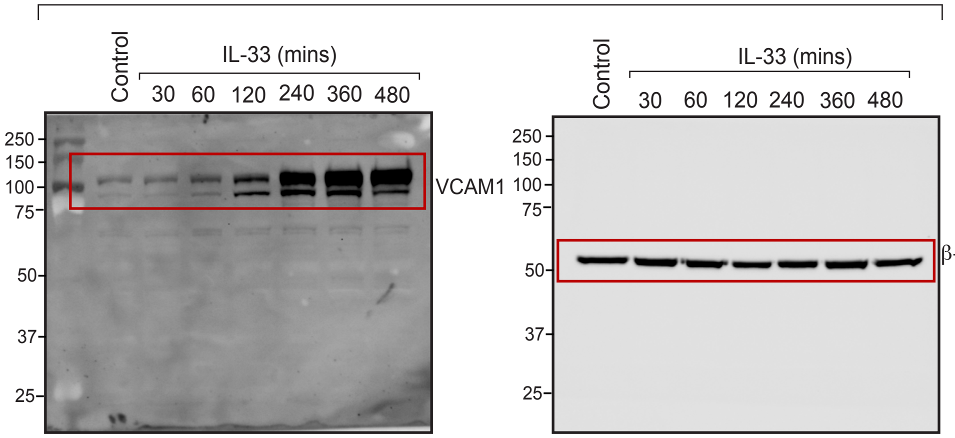

Figure 1e

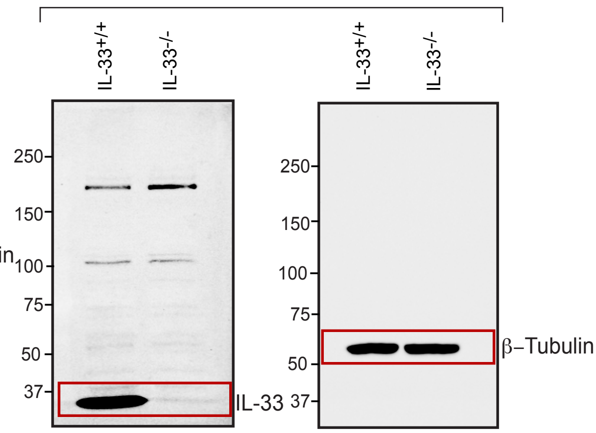

Figure 1f

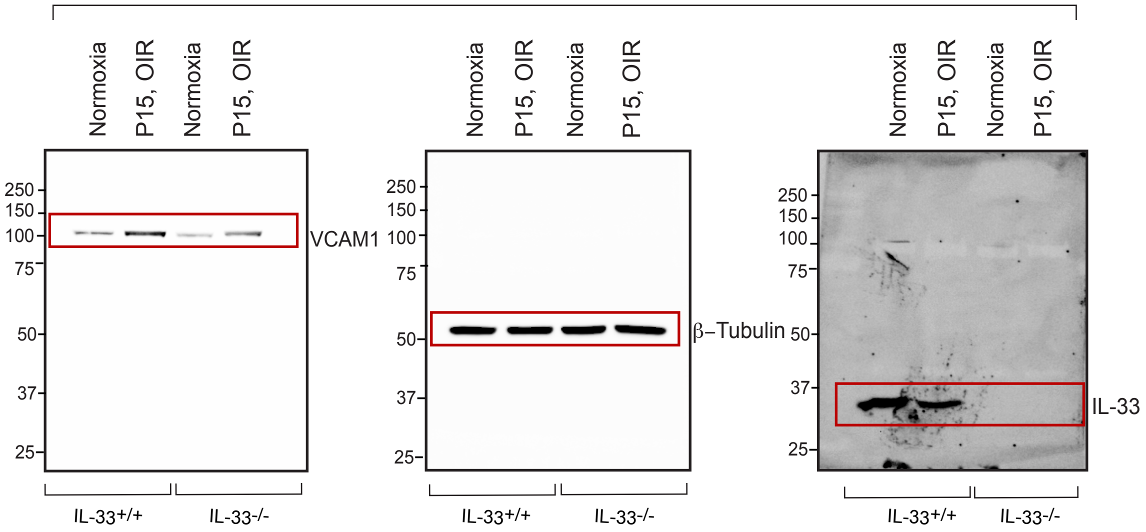

Supplementary Figure 2: Scans of immunoblots presented in Figure 1g

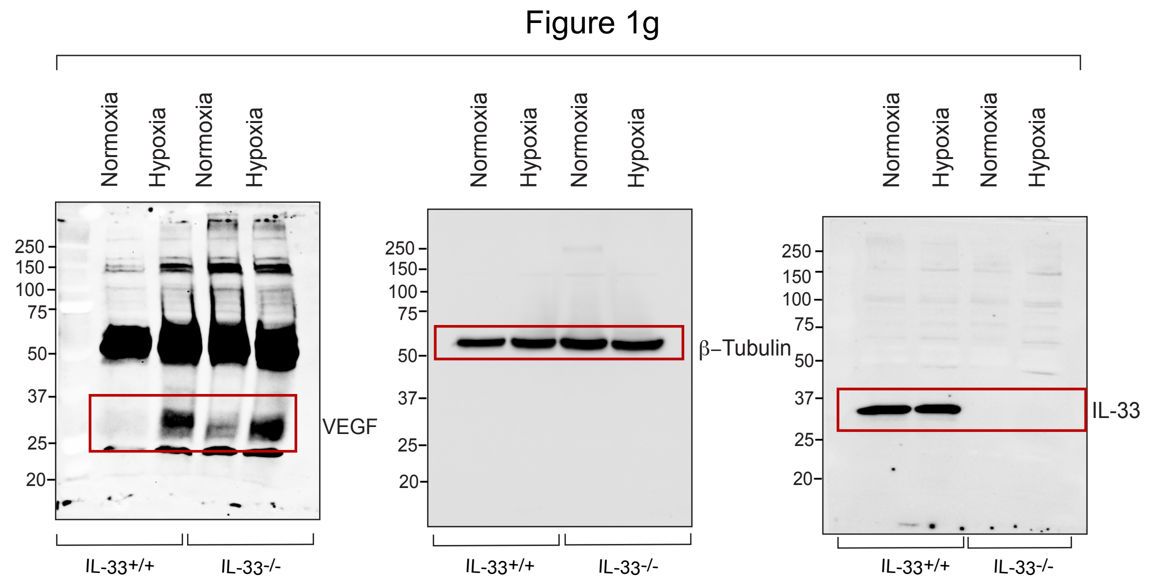

Supplementary Figure 3: Scans of immunoblots presented in Figure 2a &2f.

Figure 2a

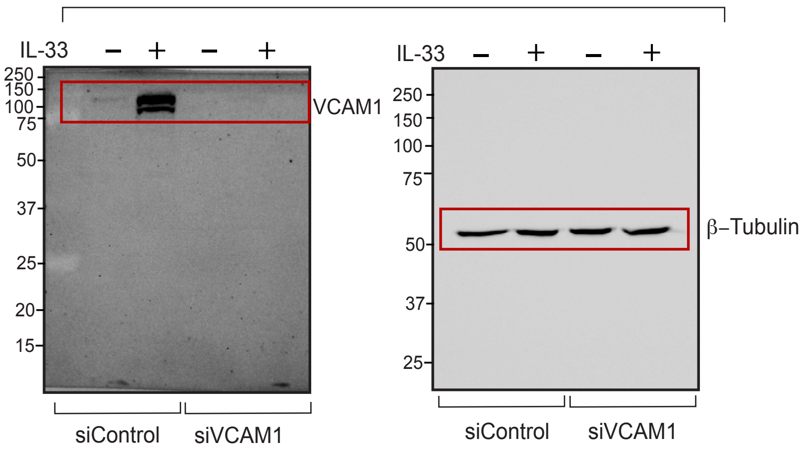

Figure 2f

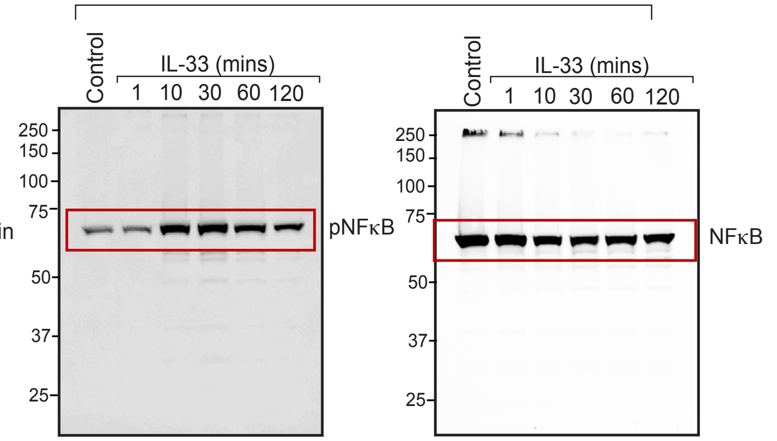

Figure 2f

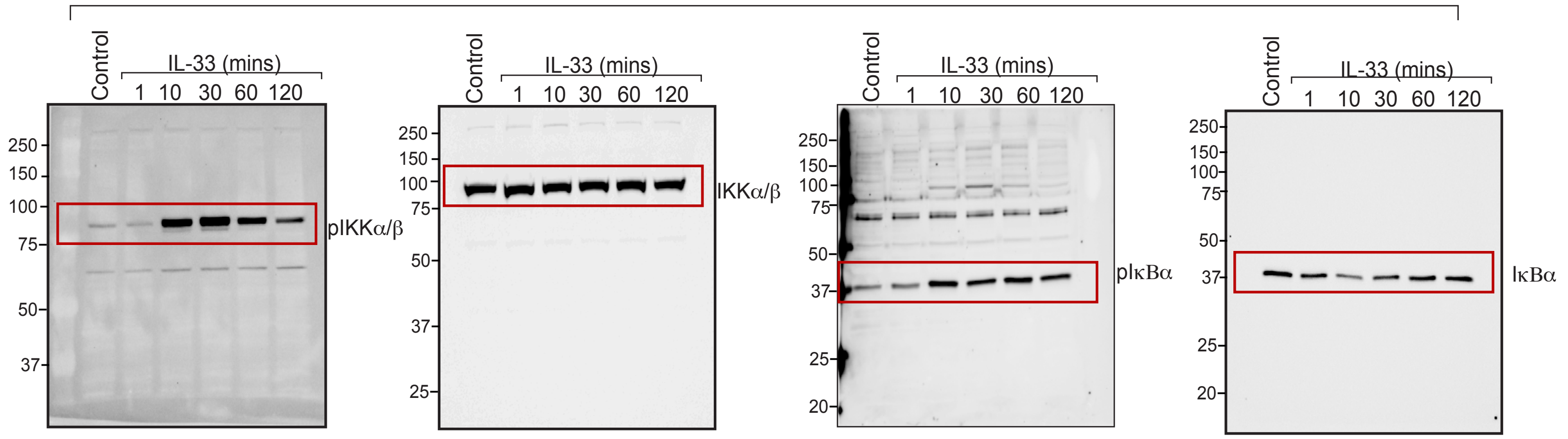

Figure 2f

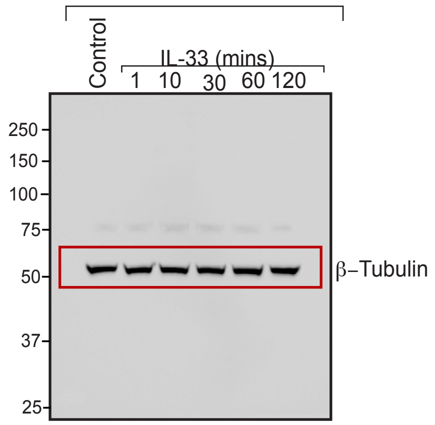

Supplementary Figure 4: Scans of immunoblots presented in Figure 2g-i, 3c & 3e.

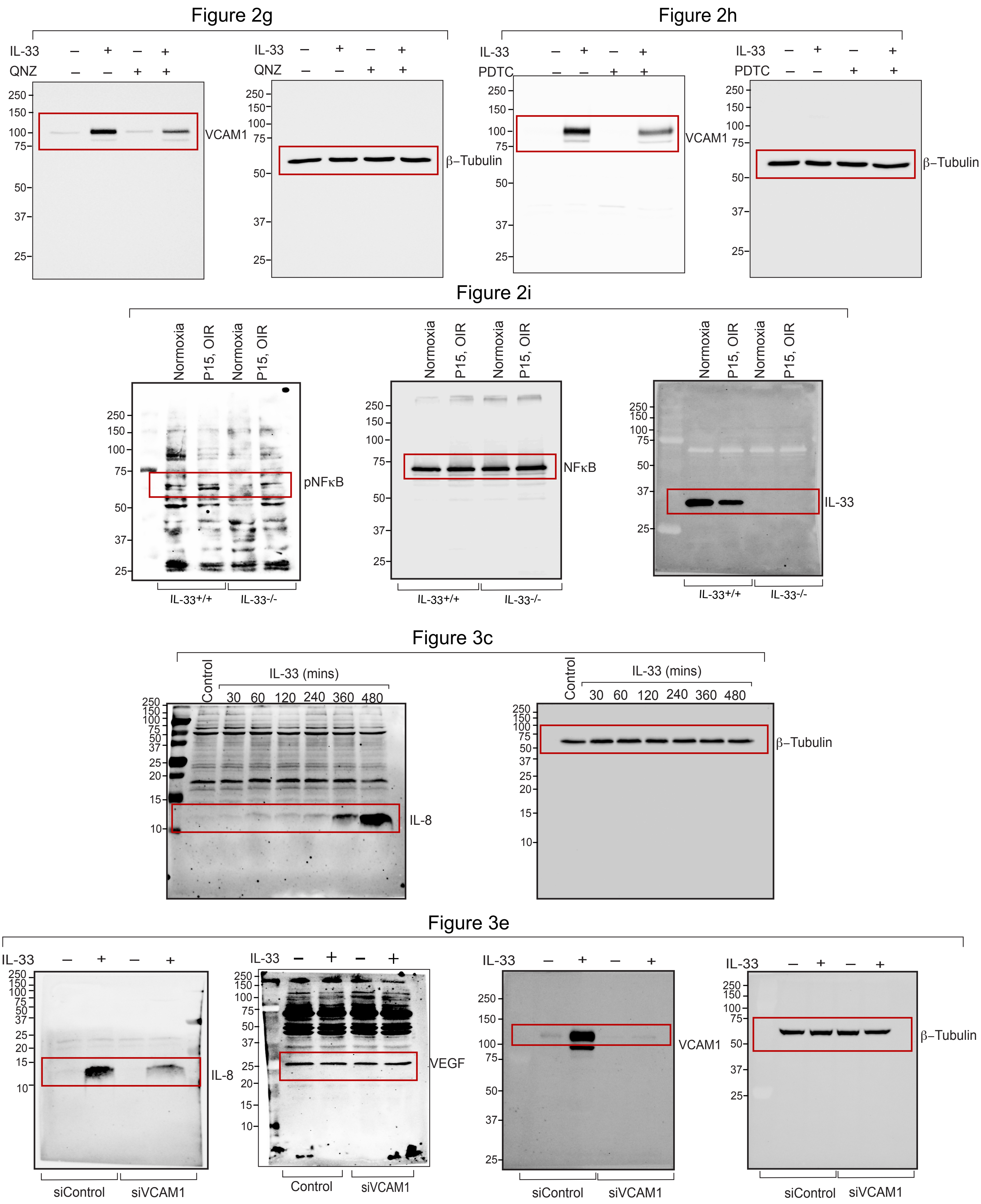

Supplementary Figure 5: Scans of immunoblots presented in Figure 3f, 3g, 4a, & 5c.

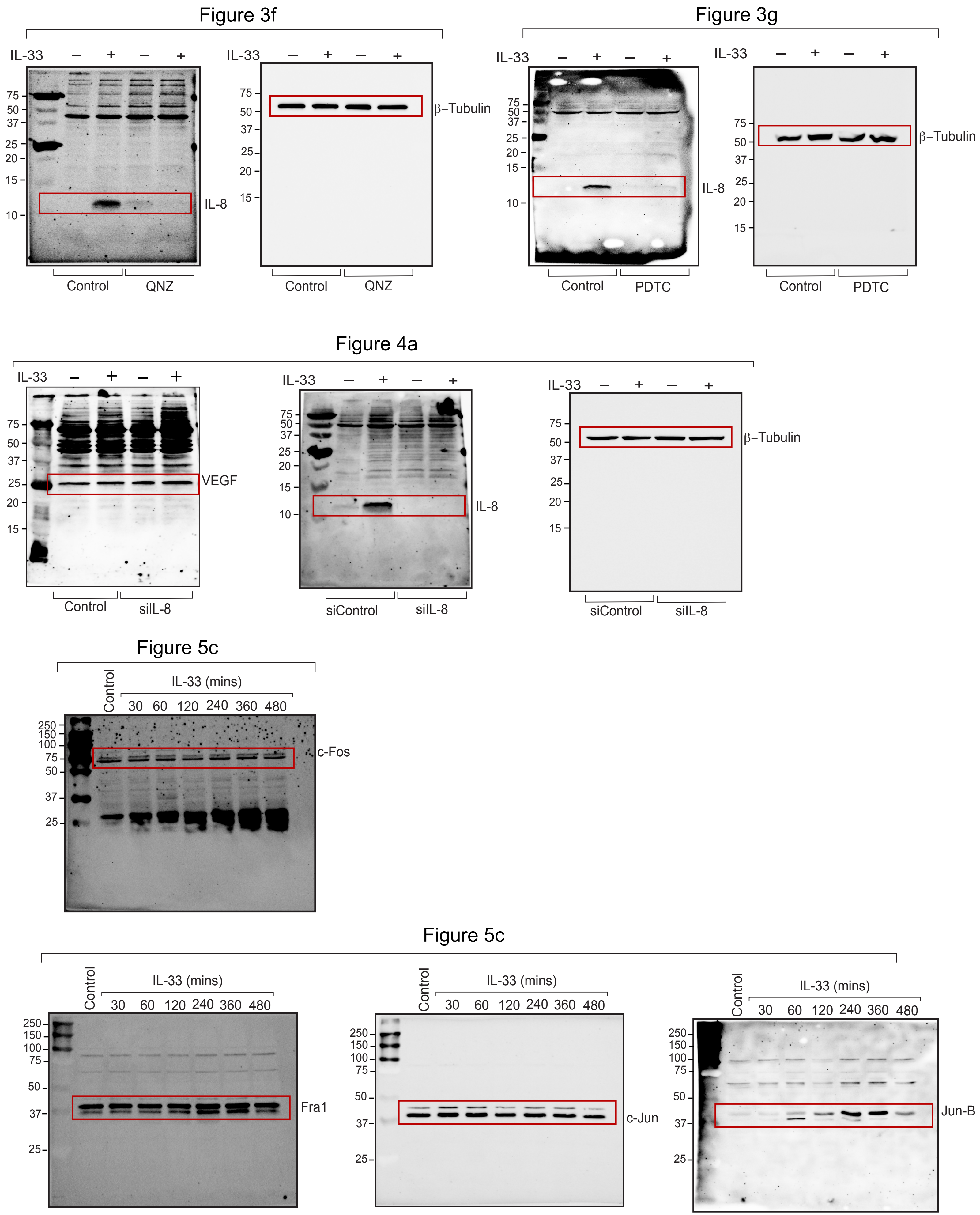

Supplementary Figure 6: Scans of immunoblots presented in Figure 5c, 5d, & 5f.

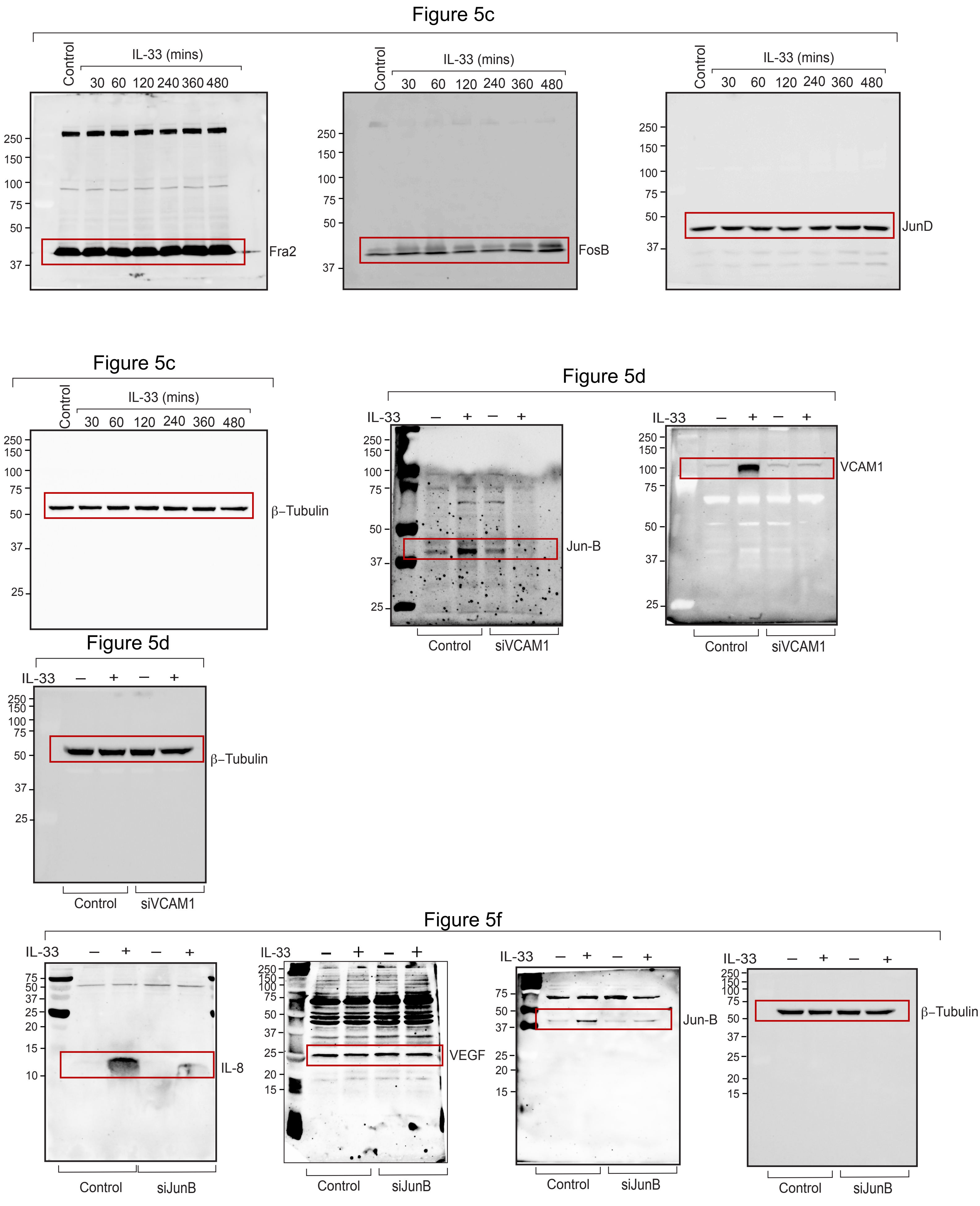

Supplementary Figure 7: Scans of immunoblots presented in Figure 5g, 5h & 6a.

Figure 5g

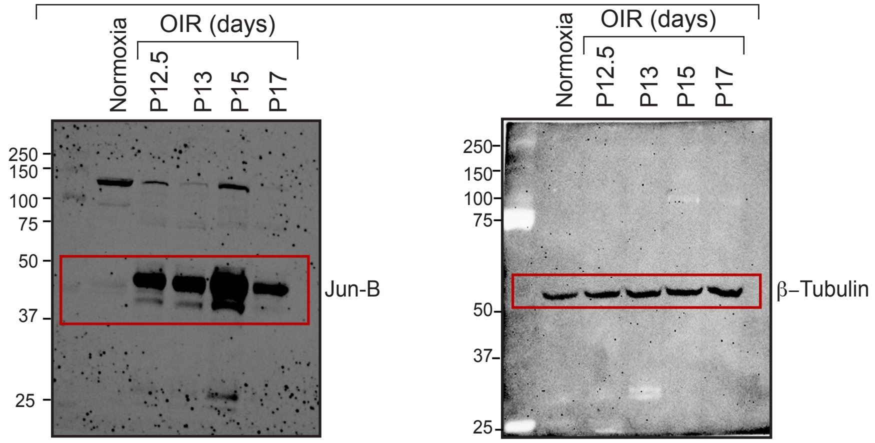

Figure 5h

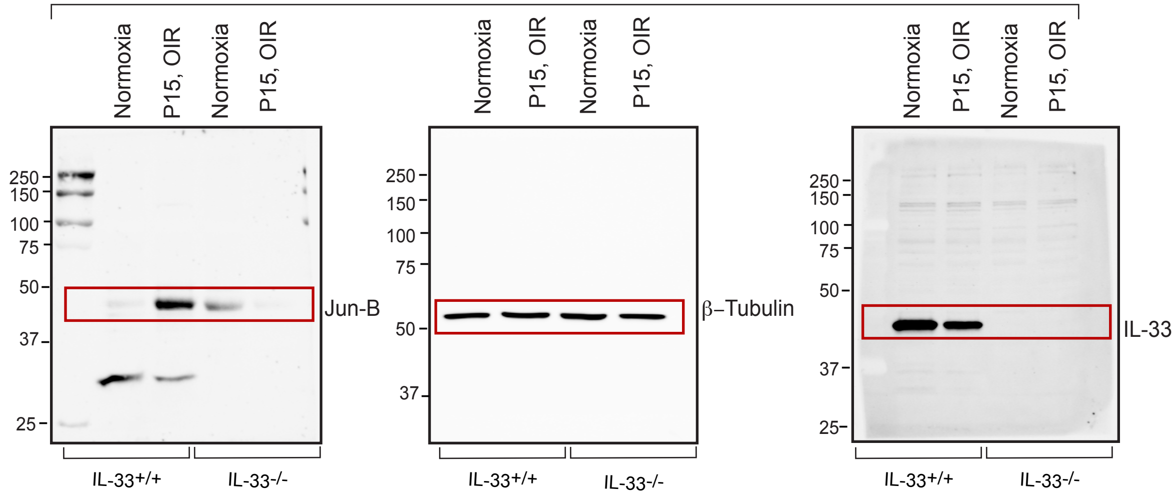

Figure 6a

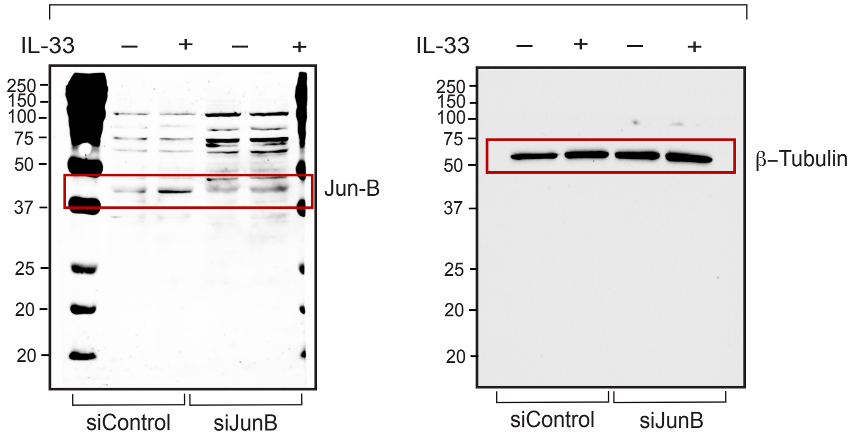

Supplementary Figure 8: Scans of immunoblots presented in Figure 8a.

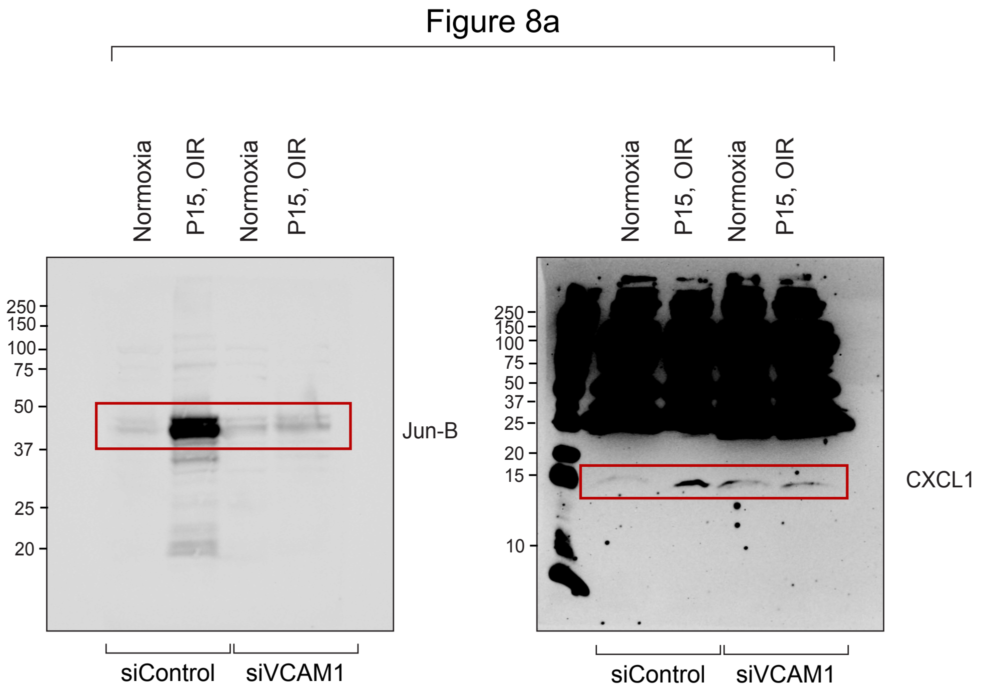

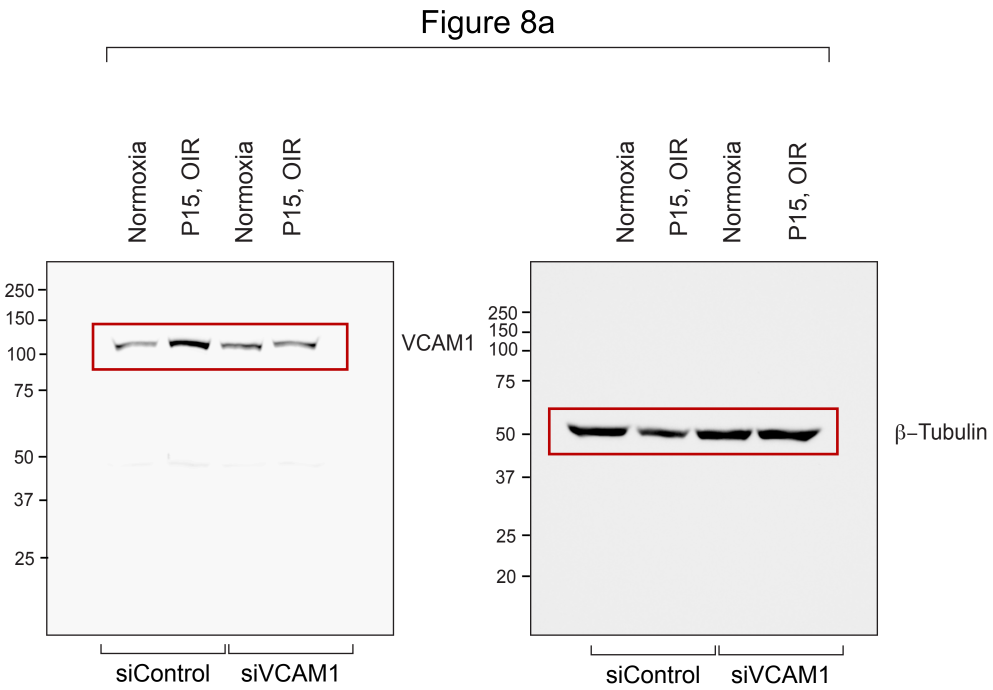

Supplement: Supplementary file 2 — Supplementary Information [file 42003_2023_4905_MOESM2_ESM.pdf]
