# Supplementary material for: Vascular cell-adhesion molecule 1 (VCAM-1) regulates JunB-mediated IL-8/CXCL1 expression and pathological neovascularization
Source: Commun Biol. 2023 May 13;6:516. doi: 10.1038/s42003-023-04905-z (PMC10183029; doi:10.1038/s42003-023-04905-z)
Supplement: Supplementary file 5 — Reporting Summary [file 42003_2023_4905_MOESM5_ESM.pdf]

nature portfolio

Corresponding author(s): *DBPR*

Last updated by author(s): *2023-4-28*

Reporting Summary

Nature Portfolio wishes to improve the reproducibility of the work that we publish. This form provides structure for consistency and transparency in reporting. For further information on Nature Portfolio policies, see our Editorial Policies and the Editorial Policy Checklist.

Statistics

For all statistical analyses, confirm that the following items are present in the figure legend, table legend, main text, or Methods section.

n/a	Confirmed	
[ ]	[X]	The exact sample size (*n*) for each experimental group/condition, given as a discrete number and unit of measurement
[ ]	[X]	A statement on whether measurements were taken from distinct samples or whether the same sample was measured repeatedly
[ ]	[X]	The statistical test(s) used AND whether they are one- or two-sided<br>*Only common tests should be described solely by name; describe more complex techniques in the Methods section.*
[ ]	[X]	A description of all covariates tested
[ ]	[X]	A description of any assumptions or corrections, such as tests of normality and adjustment for multiple comparisons
[ ]	[X]	A full description of the statistical parameters including central tendency (e.g. means) or other basic estimates (e.g. regression coefficient) AND variation (e.g. standard deviation) or associated estimates of uncertainty (e.g. confidence intervals)
[ ]	[X]	For null hypothesis testing, the test statistic (e.g. *F*, *t*, *r*) with confidence intervals, effect sizes, degrees of freedom and *P* value noted<br>*Give P values as exact values whenever suitable.*
[X]	[ ]	For Bayesian analysis, information on the choice of priors and Markov chain Monte Carlo settings
[X]	[ ]	For hierarchical and complex designs, identification of the appropriate level for tests and full reporting of outcomes
[X]	[ ]	Estimates of effect sizes (e.g. Cohen's *d*, Pearson's *r*), indicating how they were calculated

Our web collection on statistics for biologists contains articles on many of the points above.

Software and code

Policy information about availability of computer code

Data collection	Data was collected using NIH Image J version 1.53A, BD LSRII flow cytometer, NIS-Elements Advanced research software, 7300 real -time PCR system -operated SDS version 1.4, EVOS M5000 Imaging System, BIO -RAD Image Lab Software
Data analysis	*Data was analyzed using Prism 9 version 9.2.0 and Microsoft Excel version 16.57*

For manuscripts utilizing custom algorithms or software that are central to the research but not yet described in published literature, software must be made available to editors and reviewers. We strongly encourage code deposition in a community repository (e.g. GitHub). See the Nature Portfolio guidelines for submitting code & software for further information.

Data

Policy information about availability of data

All manuscripts must include a data availability statement. This statement should provide the following information, where applicable:

- Accession codes, unique identifiers, or web links for publicly available datasets
- A description of any restrictions on data availability
- For clinical datasets or third party data, please ensure that the statement adheres to our policy

All the data presented in manuscript is available on request from authors.

Field-specific reporting

Please select the one below that is the best fit for your research. If you are not sure, read the appropriate sections before making your selection.

[X] Life sciences   [ ] Behavioural & social sciences   [ ] Ecological, evolutionary & environmental sciences

For a reference copy of the document with all sections, see nature.com/documents/nr-reporting-summary-flat.pdf

Life sciences study design

All studies must disclose on these points even when the disclosure is negative.

Sample size	Sample size was calculated using standard deviation, confidence level and confidence interval.
Data exclusions	We have not excluded any data.
Replication	*All the in-vitro and in -vivo experiments are performed three times and we got the same results.*
Randomization	*The randomization is not relevant to our studies.*
Blinding	*The investigators performing in-vivo imaging were blinded to group allocation during data collection and/or analysis. For in-vitro experiments, the blinding is not relevant as the investigators were determining the role of a particular agonist on angiogensis.*

Behavioural & social sciences study design

All studies must disclose on these points even when the disclosure is negative.

Study description	*NA*
Research sample	*NA*
Sampling strategy	*NA*
Data collection	*NA*
Timing	*NA*
Data exclusions	*NA*
Non-participation	*NA*
Randomization	*NA*

Ecological, evolutionary & environmental sciences study design

All studies must disclose on these points even when the disclosure is negative.

Study description	*NA*
Research sample	*NA*

Research sample	NA
Sampling strategy	NA
Data collection	NA
Timing and spatial scale	NA
Data exclusions	NA
Reproducibility	NA
Randomization	NA
Blinding	NA

Did the study involve field work? ☐ Yes ☐ No

Field work, collection and transport

Field conditions	*NA*
Location	*NA*
Access & import/export	*NA*
Disturbance	*NA*

Reporting for specific materials, systems and methods

We require information from authors about some types of materials, experimental systems and methods used in many studies. Here, indicate whether each material, system or method listed is relevant to your study. If you are not sure if a list item applies to your research, read the appropriate section before selecting a response.

Materials & experimental systems

n/a	Involved in the study	
☐	X	Antibodies
☐	X	Eukaryotic cell lines
X	☐	Palaeontology and archaeology
☐	X	Animals and other organisms
X	☐	Human research participants
X	☐	Clinical data
X	☐	Dual use research of concern

Methods

n/a	Involved in the study	
X	☐	ChIP-seq
X	☐	Flow cytometry
X	☐	MRI-based neuroimaging

Antibodies

Antibodies used	*The antibodies used are listed at the end of this form due to space shortage.*
Validation	*Validation of each primary antibody for the species and application is provided on the manufacturer's website with relevant citations.*

Eukaryotic cell lines

Policy information about cell lines

Cell line source(s)	We used Primary Human Retinal Microvascular Endothelial Cells and obtained it from Cell-Systems (ACBRI-181, Kirkland, WA)

Authentication	*The authentication of primary cells is provided by Cell-Systems (Kirkland, WA).*
Mycoplasma contamination	*The primary cells were tested negative for mycoplasma contamination.*
Commonly misidentified lines (See ICLAC register)	*NO*

Palaeontology and Archaeology

Specimen provenance	NA
Specimen deposition	NA
Dating methods	NA

☐ Tick this box to confirm that the raw and calibrated dates are available in the paper or in Supplementary Information.

Ethics oversight	NA

Note that full information on the approval of the study protocol must also be provided in the manuscript.

Animals and other organisms

Policy information about studies involving animals; ARRIVE guidelines recommended for reporting animal research

Laboratory animals	*We used C57BL6 mice, both male and female, age:12 days to 17 days old.*
Wild animals	*NO*
Field-collected samples	*NO*
Ethics oversight	IACUC committee of Wayne State University

Note that full information on the approval of the study protocol must also be provided in the manuscript.

Human research participants

Policy information about studies involving human research participants

Population characteristics	*NA*
Recruitment	*NA*
Ethics oversight	*NA*

Note that full information on the approval of the study protocol must also be provided in the manuscript.

Clinical data

Policy information about clinical studies
All manuscripts should comply with the ICMJE guidelines for publication of clinical research and a completed CONSORT checklist must be included with all submissions.

Clinical trial registration	*NA*
Study protocol	*NA*
Data collection	*NA*
Outcomes	*NA*

Dual use research of concern

Policy information about dual use research of concern

Hazards

Could the accidental, deliberate or reckless misuse of agents or technologies generated in the work, or the application of information presented in the manuscript, pose a threat to:

No	Yes	
X	☐	Public health
X	☐	National security
X	☐	Crops and/or livestock
X	☐	Ecosystems
X	☐	Any other significant area

Experiments of concern

Does the work involve any of these experiments of concern:

No	Yes	
X	☐	Demonstrate how to render a vaccine ineffective
X	☐	Confer resistance to therapeutically useful antibiotics or antiviral agents
X	☐	Enhance the virulence of a pathogen or render a nonpathogen virulent
X	☐	Increase transmissibility of a pathogen
X	☐	Alter the host range of a pathogen
X	☐	Enable evasion of diagnostic/detection modalities
X	☐	Enable the weaponization of a biological agent or toxin
X	☐	Any other potentially harmful combination of experiments and agents

ChIP-seq

Data deposition

☐ Confirm that both raw and final processed data have been deposited in a public database such as GEO.

☐ Confirm that you have deposited or provided access to graph files (e.g. BED files) for the called peaks.

Data access links<br>*May remain private before publication.*	*NA*
Files in database submission	*NA*
Genome browser session<br>(e.g. UCSC)	*NA*

Methodology

Replicates	*NA*
Sequencing depth	*NA*
Antibodies	*NA*
Peak calling parameters	*NA*
Data quality	*NA*
Software	*NA*

Flow Cytometry

Plots

Confirm that:

- [ ] The axis labels state the marker and fluorochrome used (e.g. CD4-FITC).
- [ ] The axis scales are clearly visible. Include numbers along axes only for bottom left plot of group (a 'group' is an analysis of identical markers).
- [ ] All plots are contour plots with outliers or pseudocolor plots.
- [ ] A numerical value for number of cells or percentage (with statistics) is provided.

Methodology

Sample preparation	
Instrument	
Software	
Cell population abundance	
Gating strategy	

- [ ] Tick this box to confirm that a figure exemplifying the gating strategy is provided in the Supplementary Information.

Magnetic resonance imaging

Experimental design

Design type	*NA*
Design specifications	*NA*
Behavioral performance measures	*NA*

Acquisition

Imaging type(s)	*NA*
Field strength	*NA*
Sequence & imaging parameters	*NA*
Area of acquisition	

Diffusion MRI

- [ ] Used
- [ ] Not used

Preprocessing

Preprocessing software	*NA*
Normalization	*NA*
Normalization template	*NA*
Noise and artifact removal	*NA*

Volume censoring | NA

Statistical modeling & inference

Model type and settings | NA

Effect(s) tested | NA

Specify type of analysis: ☐ Whole brain ☐ ROI-based ☐ Both

Statistic type for inference
(See Eklund et al. 2016)

Correction | NA

Models & analysis

n/a	Involved in the study
☐	☐ Functional and/or effective connectivity
☐	☐ Graph analysis
☐	☐ Multivariate modeling or predictive analysis

Functional and/or effective connectivity | NA

Graph analysis | NA

Multivariate modeling and predictive analysis | NA

ANTIBODIES LIST

S.No.	Antibody Name	Catalog No.	Lot#	Application	Dilution	Manufacturer
1.	Anti-IL-8	Ab235584	GR3357096-6	WB	1:1000	Abcam
2.	Anti-CXCL1	Ab86436	GR3414550-1	WB	1:1000	Abcam
3.	Anti- VCAM-1	39036	1	WB	1:1000	Cell Signaling Inc.
4.	Anti-β-tubulin	2128	11	WB	1:1000	Cell Signaling Inc.
5.	Anti-P-NFkB p65	3033	17	WB	1:1000	Cell Signaling Inc.
6.	Anti-NFkB	8242	16	WB	1:1000	Cell Signaling Inc.
7.	Anti-P-IKK α/β	2697	19	WB	1:1000	Cell Signaling Inc.
8.	Anti-IKKβ	8943	5	WB	1:1000	Cell Signaling Inc.
9.	Anti-P-IkBα	2859	18	WB	1:1000	Cell Signaling Inc.
10.	Anti-IkBα	4814	17	WB	1:1000	Cell Signaling Inc.
11.	Anti-FosB	2251	3	WB	1:1000	Cell Signaling Inc.
12.	Anti-Fra2	19967	2	WB	1:1000	Cell Signaling Inc.
13.	JunD	5000	1	WB	1:1000	Cell Signaling Inc.
14.	Goat anti-IL-33	AF3626	YJE0820111	WB	1:1000	R&D Systems
15.	Anti-VCAM1	Sc-13160	A2721	WB	1:500	Santa Cruz Biotech
16.	Anti- ICAM1	Sc-8439	D2621	WB	1:500	Santa Cruz Biotech
17.	Anti-Jun-B	Sc-8051	D0821	WB	1:500	Santa Cruz Biotech
18.	Anti-Fra1	Sc-28310	G2921	WB	1:500	Santa Cruz Biotech
19.	Anti Fos/c-Fos	Sc-166940	H1721	WB	1:500	Santa Cruz Biotech
20.	Anti-c-Jun	Sc-74543	F1621	WB	1:500	Santa Cruz Biotech
21.	Anti-VEGF	Sc-57496	JH121	WB	1:500	Santa Cruz Biotech

siRNA sequences

1. **human siVCAM-1**
   sense sequence(5’->3’): GGAGUUAAUUUGAUUGGGAtt
   antisense sequence(5’->3’): UCCCAAUCAAAUUAACUCCtt
2. **mouse siVCAM-1**
   sense sequence(5’->3’): CCAUUGAAGAUACCGGGAAtt
   antisense sequence(5’->3’): UUCCCGGUAUCUUCAAUGGtg
3. **Human siJunB**
   sense sequence(5’->3’): CUCUCUACACGACUACAAAtt
   antisense sequence(5’->3’): UUUGUAGUCGUGUAGAGAGag
4. **Human siIL-8**
   sense sequence(5’->3’): GAACUUAGAUGUCAGUGCAtt
   antisense sequence(5’->3’): UGCACUGACAUCUAAGUUCtt

Primers used for IL-8 Promoter cloning

forward: 5′- TACGGGGTACCCACCAAATTGTGGAGCTTCAG -3′
reverse: 5′- CGTCAGCTAGCGCTTACCTTCACACAGAGC-3′

Cell Culture: The HRMVECs were purchased from Applied Cell Biology Research Institute (ACBRI 181, Kirkland, WA).
